# Measles mimicking HIV seroconversion syndrome: a case report

**DOI:** 10.1186/1752-1947-4-41

**Published:** 2010-02-06

**Authors:** Mahua T Chatterjee, Margaret Coleman, Gary Brook, Daniel McCrea

**Affiliations:** 1 Central Middlesex Hospital, Avery Jones Postgraduate Medical Centre, Department of Medicine, Acton Lane, Park Royal, London NW10 7NS, UK

## Abstract

**Introduction:**

Measles is on the rise in the United Kingdom and must be considered in the differential diagnosis of any patient presenting with fever and rash. As a highly infectious disease, identified patients must be isolated in the hospital setting.

**Case presentation:**

A 28-year-old Polish woman presented ill to the accident and emergency department of a district general hospital. She had painful genital ulceration, oral soreness, fever, and a facial rash. She became hypoxic within 24 hours of presentation and began to tire, thus requiring noninvasive ventilation. Her respiratory symptoms were out of proportion to the findings of her chest radiograph, which remained virtually normal. Human immunodeficiency virus seroconversion syndrome complicated by *Pneumocystis carinii *pneumonia was high among the differential diagnoses. She was given cotrimoxazole, high-dose steroids, broad spectrum antibiotics, and anti fungal cover.

Human immunodeficiency virus polymerase chain reaction came back as negative and her symptoms resolved within 10 days of presentation. She was taken off all treatment and discharged home feeling well. Serological measles was confirmed as part of a viral screen, but its clinical suspicion was low.

**Conclusion:**

The presentation of measles in this patient was unique and atypical. With its incidence rising in the United Kingdom, measles must be increasingly considered as a differential diagnosis in patients presenting with fever and rash.

## Introduction

Measles is a highly communicable acute disease that is caused by the airborne transmission of a paramyxovirus. In its typical form it is characterized by high fever, cough, coryza and conjunctivitis. Koplik's spots are rarely seen but are pathognomonic of disease. The characteristic rash appears several days after the onset of fever. The rash is maculopapular and erythematous, which spreads from the head to the torso and the extremities.

Vaccination against measles, together with improvements in the socio-economic conditions of the population, as well as improved clinical care, has reduced the high mortality rate associated with measles in many countries [1]. In the developed world, the mortality rate from measles among the immunocompetent remains low which is estimated at 1 per 1000 cases. This rises to 100 per 1000 deaths in developing countries, and 300 deaths per 1000 cases in immunocompromised patients. However, the incidence of measles is rising in the United Kingdom and in Europe [2]. This leads to concerns that endemic measles may reemerge. This is largely attributable to the vaccination controversy regarding a potential link between the combined measles, mumps and rubella (MMR) vaccine and autism. Similarly, vaccination coverage rates in many European countries have never reached the target >90% of the population [3].

With the reappearance of measles in the United Kingdom among the unvaccinated population, or in patients where the vaccine has failed to work, it must be increasingly thought of as a differential diagnosis in any patient presenting with a fever and maculopapular rash.

As with most diagnostic challenges in medicine, measles can present atypically. Here we present an unusual case of measles which was initially identified as human immunodeficiency virus (HIV) seroconversion syndrome.

## Case presentation

A 28-year-old Polish woman who has been residing in the United Kingdom for four years presented to the accident and emergency department of a district general hospital with severe ulceration of the perineal area, dysuria, soreness of the mouth, fever, non-productive cough, and a facial rash. Her illness started two weeks prior to presentation with a flu-like illness and sore throat for which she was prescribed with antibiotics. Three days into the illness, she developed severe burning and itching of her genital region. Antifungal topical treatment and appropriate antibiotic therapy for a concurrent urinary tract infection provided minimal relief. She further developed high fever, vomiting, and a facial rash.

Her medical history included von Willibrand's disease and a caesarean section two years prior to presentation. She had no relevant drug history or sexual history.

On examination our patient was unwell with a temperature of 38°C, heart rate of 110 beats per minute, blood pressure of 105/60, and initial oxygen saturations of 100% on air.

She had crepitations at the base of her left lung, tenderness on palpation at the right upper quadrant of her abdomen, and an ill-defined rash over her face and neck. There was some erythema at the back of her mouth and a white coating resembling candida. She had mild injection of both eyes.

The most marked findings were of vulval and perineal erythema and excoriations with discrete ulceration. The associated pain was so severe it prevented her from moving her legs freely.

Further investigations revealed that she had a lymphopenia of 0.26 × 10^9^/L (NR = 1.10 to 4.80) and a C-reactive protein (CRP) level of 49 (NR = <5). Meanwhile, abnormal liver function tests revealed an alanine transaminase (ALT) level of 342I U/L (NR = 0 to 55) and alkaline phosphatase (ALP) level of 185 IU/L (NR = 40 to 150).

With these initial findings of genital ulceration, oral soreness and candidiasis, fever, and rash, we sought an infectious disease opinion. The differential diagnosis at this stage included human immunodeficiency virus (HIV), herpes simplex virus, and noninfective causes such as Bechet's, systemic lupus erythematosus, and systemic vasculitis. Reiter's syndrome was also considered as it can present without arthritis in women.

Tests for viral screen (including measles IgM and IgG), anti-streptolysin O (ASO) titre, HIV, PCR, complement levels, autoimmune screen, and immunoglobulin levels were requested. Our patient, meanwhile, was initially treated with intravenous acyclovir and high-dose prednisolone.

**Figure 1:**
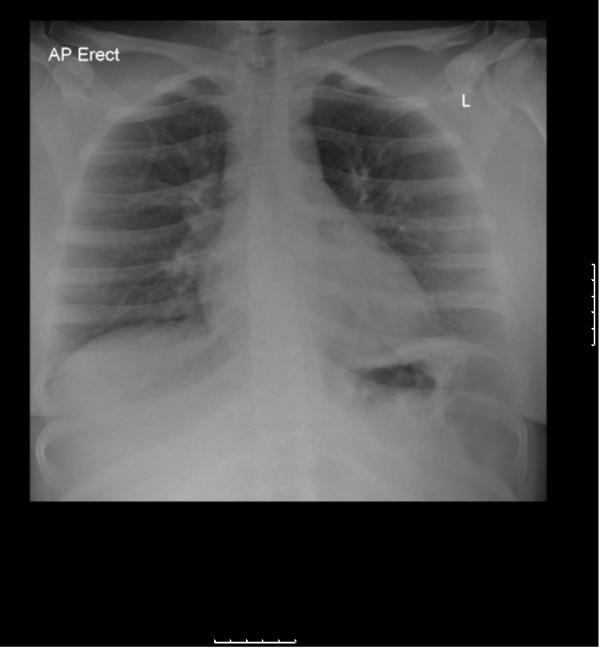
**Severe hypoxia requiring noninvasive ventilation is not explained by this relatively normal chest X-ray**.

Within 20 hours of presentation she developed type 1 respiratory failure with a pO2 of 9.5 KPa on 10 liters of oxygen. She began to tire and required noninvasive ventilation. Her respiratory symptoms were out of proportion to the changes exhibited in her chest X-ray (minimal consolidation at her left lung base, see Figure 1), although clinically she began to develop bronchial breathing at her left lung base.

The most likely diagnosis at this stage was HIV seroconversion complicated by *Pnemocystis carinii *pneumonia. Another possibility was of an atypical but severe bacterial infection. She was treated with intravenous broad spectrum antibiotics (imipenem and teicoplanin) and treatment doses of cotrimoxazole. Her steroids were also changed to high-dose methylprednisolone.

On day 4 of admission her facial rash had resolved and her oxygenation began to improve (PO2 13.1 Kpa, Fio2 at 40%). Meanwhile, her blood tests revealed negative HIV PCR, negative ASO titre, normal complement levels, normal immunoglobulin levels, and negative autoimmune screen. Her steroid medication was slowly weaned and the antibiotics, including cotrimoxazole, were stopped.

Her symptoms had completely resolved by day 10 of admission. It seemed likely that she had an atypical viral infection. Elevated serum titres of IgM and IgG antibodies to measles were subsequently demonstrated, thus confirming a diagnosis of acute measles. The patient is now well.

A vaccination history of our patient revealed that she had completed all her childhood vaccines in Poland.

## Discussion

Measles meets the criteria for diseases that can be eradicated and yet remains endemic worldwide. The World Health Organization estimates that measles causes 454,000 deaths annually [4]. Most of these deaths occur in Africa and in Southeast Asia.

In the United Kingdom, measles associated death remains low: 2006 saw the first measles death since 1992. However, the incidence of this disease is rising with localized outbreaks in unvaccinated children and adults. This is largely attributable to low vaccine uptake rates surrounding a controversy over the safety of the vaccine in the late 1990s. There is now much evidence, however, to refute such a link [5].

Localized outbreaks are often initiated by an imported case from Europe or elsewhere. In the United States, for example, measles was declared eliminated in 2000. In 2005, however, an unvaccinated 17-year-old girl returning from Romania was found to be incubating the disease. This case was responsible for the largest recent outbreak of measles in the United States [6].

With the rising incidence of measles, it must be considered increasingly as a differential diagnosis in any patient presenting with a fever and a maculopapular rash. A careful immunization history should be included in the medical clerking.

The patient we discussed in this case report had received one dose of the measles vaccine in Poland when she was a baby. There was no record of a second dose being given and she had never since been tested for serological evidence of immunity. A single dose vaccine schedule, even with a coverage of 100% of the population, allows the gradual accumulation of a cohort of susceptible people, while a second dose confers immunity to 99% of those vaccinated [3]. Nevertheless, vaccination failure has also been recorded [3].

This case illustrates the diagnostic difficulties of atypically presenting measles and emphasizes that the disease course can be moderately severe in adults. The main presenting complaint in this case was genital ulceration and severe perineal pain.

Interestingly, the measles virus infects epithelial cells of the host after airborne transmission, which may then lead to a replication in the urinary tract [7]. This could explain the symptoms of dysuria. However, to the best of our knowledge, there have been no reported cases in the literature of measles causing genital ulceration.

While the oral soreness and white coating at the back of our patient's mouth resembled candida, it is more likely that this was pathognomonic Koplik's spots. Their location was unusual as they are more commonly found on the buccal mucosa. Koplik's spots also normally appear in the prodromal period of illness and disappear before the onset of the measles rash. Koplik's spots have less commonly been reported 2 to 3 days after the onset of rashes.

Our patient's level of hypoxia was highly unproportional to the minimal consolidation seen on her chest radiograph. This, together with the mucosal findings and non-specific prodrome, made HIV serconversion with concurrent PCP high on our differential list.

The diagnosis of measles was picked up as part of a viral screening test although its clinical suspicion was low. In retrospect, the pointers to measles in this case were flu-like prodrome, maculopapular rash, injection of the eyes, and Koplik's spots. The severe genital ulceration and oral soreness was a unique presentation of the disease. This focused our differential more toward diseases that involves primarily the mucous membranes.

This case highlights that measles can be of severe presentation in the young adult. While complication and mortality rates increase in adulthood, measles associated death rates are highest below the age of 1 year and lowest between the ages of 1 to 9 years.

The severe hypoxia and bronchial breathing in the context of a normal chest X-ray was likely to be measles associated pneumonitis or as a result of superimposed infection with bacteria or other viruses. Pneumonia accounts for 56% to 86% of all measles associated deaths [8].

Measles is highly infectious and clinical suspicion must remain high especially in a hospital setting because these cases need isolating. In 2006, this district general hospital had seven healthcare professionals with positive serology for measles [9], of which four required hospitalization. Screening of healthcare professionals at the time of the outbreak showed that 3% of hospital staff members were not immune to measles.

This case emphasizes the importance of considering measles as a differential diagnosis in any patient presenting with rash and fever.

## Conclusion

This case illustrates that measles can present atypically and that it can be a severe illness in young adults. With the incidence of measles rising in the United Kingdom, it must be increasingly considered as a differential diagnosis in patients presenting with fever and rash.

## Consent

Written informed consent was obtained from the patient for publication of this case report and any accompanying images. A copy of the written consent is available for review by the Editor-in-Chief of this journal.

## Competing interests

The authors declare that they have no competing interests.

## Authors' contributions

MTC prepared the manuscript. MC drafted sections of the manuscript. GB was involved in the acute assessment of the patient and proofread the manuscript. DM was involved in the acute assessment of the patient and was the physician responsible for the patient's care. He also drafted the original manuscript. All authors read and approved the final manuscript.
